# Isolated sixth cranial nerve palsy as the presenting symptom of a rapidly expanding ACTH positive pituitary adenoma: a case report

**DOI:** 10.1186/1471-2415-11-4

**Published:** 2011-01-27

**Authors:** Norman Saffra, Elizabeth Kaplow, Irina Mikolaenko, Alice Kim, Benjamin Rubin, Jafar Jafar

**Affiliations:** 1 Department of Ophthalmology, Mount Sinai School of Medicine, New York, NY, USA; 2 Department of Ophthalmology, Maimonides Medical Center, Brooklyn, New York, USA; 3 Mount Sinai School of Medicine, New York, NY, USA; 4 Department of Pathology, Division of Neuropathology, New York University, New York, NY, USA; 5 Department of Neurosurgery, New York University Langone Medical Center, New York, NY, USA

## Abstract

**Background:**

Pituitary adenoma may present with neuro-ophthalmic manifestations and, typically, rapid tumor expansion is the result of apoplexy. Herein, we present the first case of an isolated sixth cranial nerve palsy as initial feature of a rapidly expanding ACTH positive silent tumor without apoplexy.

**Case Presentation:**

A 44 year old female with a history of sarcoidosis presented with an isolated sixth cranial nerve palsy as the initial clinical feature of a rapidly expanding ACTH positive silent pituitary adenoma. The patient underwent emergent transsphenoidal hypophysectomy for this rapidly progressive tumor and subsequently regained complete vision and ocular motility. Despite tumor extension into the cavernous sinus, the other cranial nerves were spared during the initial presentation.

**Conclusions:**

This case illustrates the need to consider a rapidly growing pituitary tumor as a possibility when presented with a rapidly progressive ophthalmoplegia.

## Background

Pituitary adenoma, the most common cause of sellar masses after the third decade of life, may present with neuro-ophthalmic manifestations. Visual impairment is the most common presenting symptom in patients with a non-secreting adenoma as a result of chiasmal compression [1-3]. Less commonly, lateral extension of the adenoma into the cavernous sinus will often cause compression of the third cranial nerve [4], resulting in diplopia. These tumors typically grow slowly unless pituitary apoplexy - defined as acute hemorrhagic infarction of the pituitary adenoma causing increased intra-sellar pressure often resulting in acute onset of headache, nausea, vomiting, ophthalmoplegia, visual loss, and even hypopituitarism - is present [5]. Herein we present the first case of an isolated sixth cranial nerve palsy as the presenting clinical feature of a rapidly expanding ACTH positive silent tumor without apoplexy.

## Case Presentation

A 44-year-old female with past medical history significant for sarcoidosis, not currently receiving treatment, and hypertension presented to the clinic with a chief complaint of vague headaches and horizontal diplopia that worsened with left gaze of one days' duration. Neuro-ophthalmologic exam revealed an isolated, incomplete, left cranial nerve six palsy. Visual acuity was 20/20 bilaterally, Humphrey visual field testing with 24-2 testing strategy was full bilaterally, and dilated fundus examination revealed the absence of papilledema and a normal retinal periphery. There was no clinical evidence of either myasthenia gravis or thyroid ophthalmopathy.

**Figure 1:**
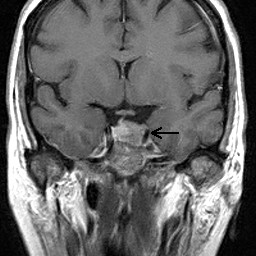
**T1 post-contrast axial MRI shows a tumor within the sella displacing the pituitary gland and infundibulum**.

**Figure 2:**
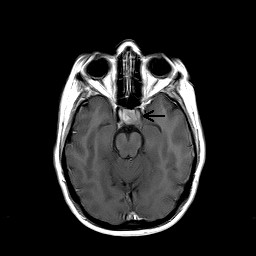
**T1 post-contrast coronal MRI shows a mass impressing on the left cavernous sinus**.

An MRI of the orbits and brain (Figures 1, 2) revealed a 1.5 × 1.9 × 1.4 cm mass within the sella, displacing the pituitary gland and infundibulum towards the right and impinging on the left cavernous sinus. The mass extended into the suprasellar cistern, but did not compress the optic chiasm. There were intra-lesional areas of increased signal intensity on T1 and T2 weighted sequences that likely represented minimal hemorrhage, insufficient to qualify as apoplexy. There was no evidence of intracranial sarcoidosis.

The patient was immediately admitted to the neurosurgical service for preoperative studies and scheduled for urgent transsphenoidal hypophysectomy. Preoperative and systemic work up was initiated, inclusive of complete blood count, metabolic profile, coagulation studies, sedimentation rate, thyroid function tests, acetylcholine receptor antibodies both binding and blocking, & angiotensin converting enzyme. All tests were within normal limits aside from an elevated ACE, which was unchanged from previous examination. Review of history and physical exam revealed no clinical evidence of hypercortisolemia, hyperprolactinemia, or menstrual dysfunction. Evaluation of the pituitary hormone status of tumor, inclusive of ACTH, cortisol, prolactin, IGF-1, LH, and FSH, was scheduled but could not be completed due to rapid clinical deterioration.

The patient remained stable over the next 36 hours, at which point she developed a left-sided, severe, retro-orbital headache. Over the next four hours the patient developed acute left sided ptosis and proptosis with complete ophthalmoplegia and decreased visual acuity, findings consistent with complete cavernous sinus involvement. An emergent head CT was then obtained which demonstrated an enlarging mass in the sella without evidence of intra- or extra-lesional hemorrhage. Final dimensions of the tumor on this non-contrast head CT were 1.5 × 1.3 × 2.4 cm, overall a significant increase in size from the measured dimensions of 1.5 × 1.9 × 1.4 cm found on initial MRI especially given that MRI has been shown to provide better evaluation of pituitary macroadenomas [6,7].

**Figure 3:**
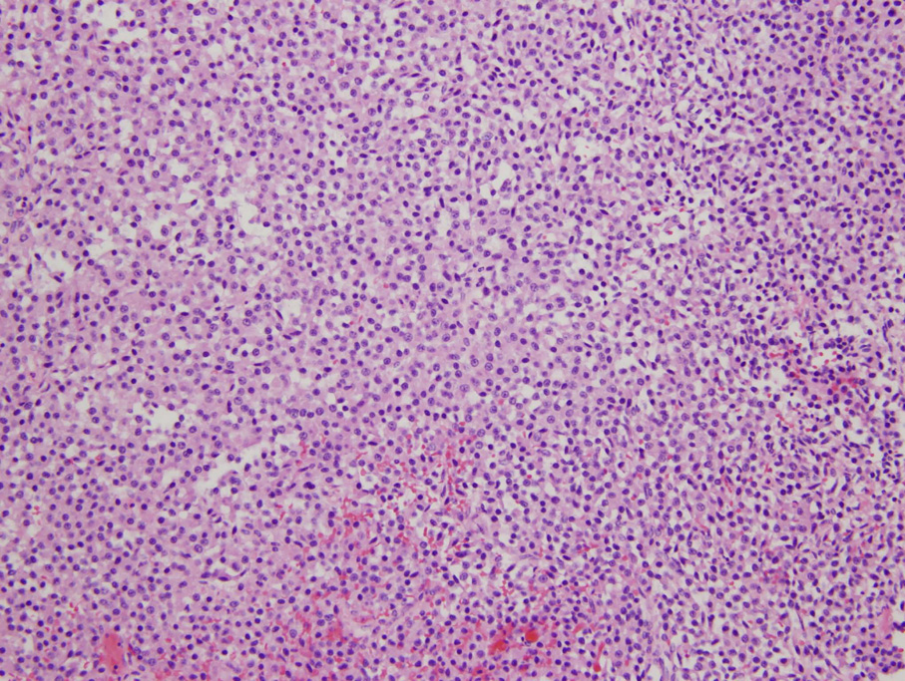
**Histopathology revealed pituitary adenoma composed of monomorphous cells with amphophilic cytoplasm (hematoxylin and eosin, X20)**.

**Figure 4:**
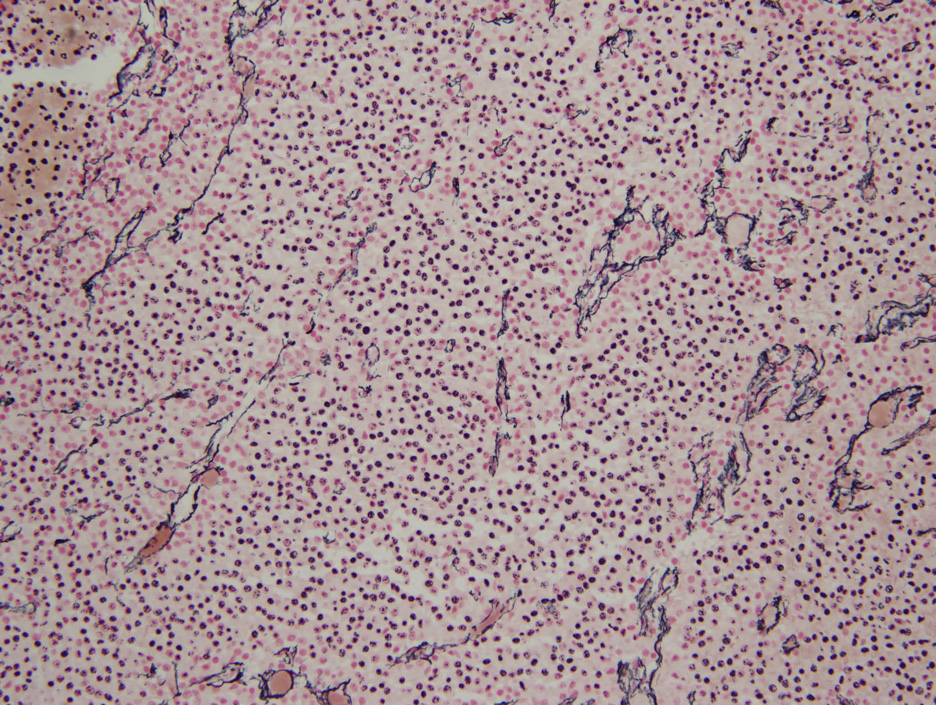
**Reticulin special stain demonstrates loss of the usual acinar pattern supporting the diagnosis of pituitary adenoma (hematoxylin and eosin, X20)**.

**Figure 5:**
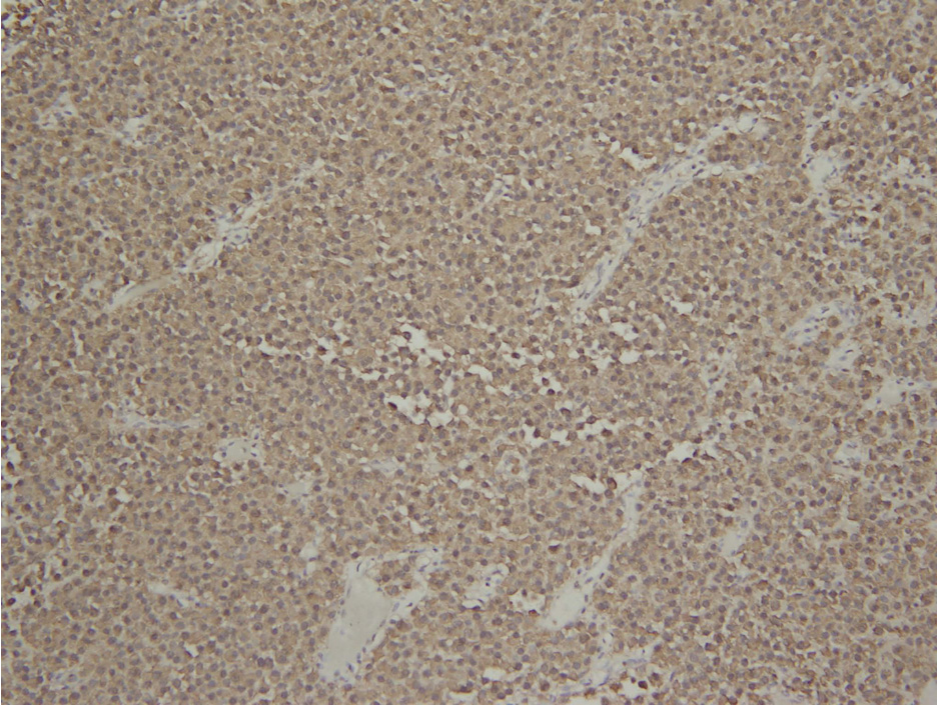
**The tumor is diffusely positive for ACTH immunostain (hematoxylin and eosin, X20)**.

The patient underwent emergent transsphenoidal hypophysectomy for this rapidly progressive tumor. Intraoperatively, the tumor was found to have clinical extension into the cavernous sinus. Pathologic examination of the mass (Figures 3, 4, 5) demonstrated a 1.5 × 1.0 × 0.3 cm pituitary adenoma positive for ACTH immunostain. No frank hemorrhage or necrosis was noted. Post-operatively the patient was placed on intravenous Decadron, which was tapered over 10 days. She immediately regained visual acuity and the VIth nerve palsy resolved over the subsequent two months. Post-operative scans, however, continued to demonstrate tumor presence and growth and the patient underwent a second resection one month following the initial surgery, followed by stereotactic Gamma knife radiation seven months later. Now, 5 months post her last procedure, she remains free of symptoms with complete ocular motility, 20/20 visual acuity, and full visual fields. There have been no changes in her reported symptoms of sarcoidosis.

## Conclusions

The cavernous sinus contains the carotid artery as well as the oculomotor, trochlear, ophthalmic and maxillary divisions of the trigeminal, and abducens nerves. Extraocular palsy generally indicates compression of the cavernous sinus wall or direct extension of the pituitary adenoma into the cavernous sinus [3,8]. The incidence of ocular palsy occurring with pituitary tumors has been reported to be between 4.6 and 32% [9-12]. Most commonly affected is the oculomotor nerve; rarely is the abducens nerve involved [3,13]. A retrospective review of 64 patients hospitalized with pituitary adenoma [9] found that a defect in ocular movement was present in 14% of patients, with oculomotor nerve involvement in 78% of cases. The sixth cranial nerve runs lateral to the internal carotid artery, but medial to the third, fourth, and first and second divisions of the fifth cranial nerves which run superior to inferior within the lateral dural border of the cavernous sinus. It is therefore more often spared because of its more sheltered position within the sinus [14]. Our patient is unusual in that initially the sixth cranial nerve paresis was in isolation.

Despite the patients past medical history being positive for sarcoidosis, there was no evidence of neurosarcoidosis in this case. Overall, 25-60% of patients with systemic sarcoidosis will develop ocular symptoms, while 5% will develop neurosarcoidosis. Cranial nerve neuropathies are the most frequent neurological manifestation; cranial nerves II, III, IV, V, VI, and VII may be affected individually or in combination [15-17].

The rapid expansion of the tumor secondary to tumor growth and not apoplexy, as evidenced by the lack of acute hemorrhage observed on imaging and subsequently on pathological examination, in this ACTH positive silent tumor is highly unusual. In a series of non-operated patients, the mean increase in tumor diameter was 0.6 mm per year [5]. Furthermore, in a five year period only half of macroadenomas demonstrated any growth [18]. There has been evidence to suggest that silent corticotroph adenomas, which are histologically and immunocytologically distinct from clinically non-functioning adenomas, have a higher frequency of aggressiveness and recurrence. The most common finding associated with these silent corticotroph tumors is rapidly progressive visual field defects. A study of 23 cases of silent pituitary corticotroph adenomas [20] found that these tumors were more likely to be macroadenomas, undergo hemorrhagic infarction, present with symptoms due to mass effect, and have a high rate of recurrence. However, increased rates of aggressive behavior in ACTH-positive tumors could not be demonstrated in other studies [18-21]. This case suggests that pituitary neoplasms that demonstrate rapid expansion as a result of tumor growth may require different treatment paradigms than typically behaving lesions.

## Competing interests

The authors declare that they have no competing interests; there was no grant support or research funding and no proprietary interests in the materials described

## Authors' contributions

AK, NS, JJ, and BR treated the patient and in doing so acquired the case data; they were also involved with drafting of the manuscript. IM interpreted the pathological specimens and was involved with drafting of the manuscript. EK assisted in data acquisition and was involved with drafting the manuscript. All authors read and approved the final manuscript.

## Pre-publication history

The pre-publication history for this paper can be accessed here:

http://www.biomedcentral.com/1471-2415/11/4/prepub
